# A Study on the Separation of Nitric Acid and Acetic Acid from Simulated Reprocessing Waste by TBP Extraction

**DOI:** 10.3390/molecules30081814

**Published:** 2025-04-17

**Authors:** Hongbin Lv, Xiao Ge, Tiansheng He, Baole Li, Tianchi Li, Hui Wang, Zhongwei Yuan, Qi Yang, Taihong Yan

**Affiliations:** China Institute of Atomic Energy, China National Nuclear Corporation, Beijing 102413, China; lvhongbin@cnnc.com.cn (H.L.); 18810987075@163.com (X.G.); hetiansheng@tju.edu.cn (T.H.); libleanty@163.com (B.L.); 15810713099@163.com (T.L.); hwihwi@126.com (H.W.); yuanzw99@163.com (Z.Y.); yqmaytree@163.com (Q.Y.)

**Keywords:** nitric acid, acetic acid, TBP, separation

## Abstract

The PUREX process is a key technology for spent fuel reprocessing, designed to selectively recover uranium and plutonium mainly through multiple chemical separation stages, minimizing high-level waste. Acetohydroxamic acid (AHA) enhances selectivity in this process but decomposes into acetic acid (HAc), which disrupts chemical equilibrium and reduces extraction efficiency. This study examines the extraction and separation of nitric acid (HNO_3_) and HAc using 30% tributyl phosphate in organic kerosene (TBP-OK) under various conditions. Results show that 30%TBP-OK preferentially extracts HAc over HNO_3_, especially in the low acid concentration range (HNO₃ < 1 mol/L, HAc < 0.2 mol/L). The selectivity coefficient drops from 3.05 in a 0.5 mol/L HNO_3_-0.1 mol/L HAc system to 2.18 in a 1 mol/L HNO_3_-0.2 mol/L HAc system. TBP forms stable 1:1 complexes with both acids, with equilibrium constants around 0.85 under typical conditions. Increasing TBP concentration enhances HNO_3_ extraction, while phase ratio adjustments improve HAc separation. A 16-stage countercurrent extraction simulation confirms that optimizing these factors effectively separates HNO_3_ and HAc, offering theoretical and technical support for refining the PUREX process.

## 1. Introduction

With the rising global demand for energy and increasingly stringent environmental regulations, nuclear energy is gaining recognition for its efficiency and environmentally friendly attributes [1,2,3,4]. The substantial volume of spent fuel produced by nuclear power plants must be effectively managed to ensure the sustainable utilization of nuclear fuel resources and the safe disposal of radioactive waste [5,6]. Spent fuel reprocessing technologies enable the recovery of fissionable materials such as uranium and plutonium while significantly reducing the volume and toxicity of high-level radioactive waste [3]. The Purex process is currently the most widely used method for spent fuel reprocessing.

In the Purex process, tributyl phosphate (TBP) is commonly used as an extractant, with kerosene serving as a diluent to form the organic phase [7,8]. This organic phase interacts with the aqueous phase to achieve the separation of uranium and plutonium based on their distribution behavior between the two phases. In recent years, acetohydroxamic acid (AHA) has been introduced as a reducing and complexing agent for plutonium to enhance the selectivity and safety of the Purex process by controlling its redox state [9]. However, AHA has a limited stability in the acidic environment and tends to decompose, producing byproducts such as acetic acid (HAc), ammonia, and carbon dioxide [10]. The accumulation of acetic acid not only alters the pH of the solution but also affects the performance of the extractant and reduces the overall extraction efficiency. Consequently, the effective removal of acetic acid and the recovery of nitric acid have become critical challenges in optimizing the PUREX process.

Traditional wastewater treatment methods [11,12], such as neutralization precipitation and evaporation concentration, often struggle to efficiently separate nitric acid and acetic acid. These techniques are typically associated with high costs, significant energy consumption, and secondary pollution. Ion exchange resins offer advantages such as low energy consumption, strong selectivity, and operational simplicity [13]. However, their limited adsorption capacity, difficulty in desorption, and degradation in performance after repeated use make them unsuitable for large-scale application. The associative properties of acetic acid in aqueous phase and the high volatility of nitric acid [14] lead to substantial energy consumption and poor separation efficiency when using the distillation method.

In contrast, liquid–liquid extraction offers a highly selective and efficient separation process by utilizing the differing solubilities of compounds in immiscible solvents [15,16,17]. It offers notable advantages, including high selectivity, excellent separation efficiency, and process simplicity. In recent years, advancements in solvent extraction technology have made liquid–liquid extraction an increasingly attractive solution for treating acidic wastewater [18,19,20,21,22]. Research has shown that using suitable extractants can effectively improve the separation efficiency of nitric acid and acetic acid, facilitating resource recovery from acidic wastewater [13,22,23,24].

Tributyl phosphate (TBP) is one of the most commonly used extractants due to its strong affinity for acidic solutes and its exceptional chemical stability in the presence of strong acids, bases, and oxidizing agents [25,26,27,28,29]. The TBP-kerosene (TBP-OK) extraction system, formed by dissolving TBP in kerosene as a diluent, is widely used for the separation of acidic substances [30,31,32]. Moreover, the use of the TBP-OK extraction system in the PUREX process eliminates the introduction of additional impurities that would require secondary separation. A. K. Shakya et al. [33] investigated the reactive extraction of acetic acid using tributyl phosphate (TBP) as the extractant with different diluents. Their results indicated that a TBP volume fraction of 40% achieved the highest extraction efficiency. Their findings demonstrated that, under suitable operating conditions, TBP exhibits excellent extraction performance for acetic acid. Additionally, in their study of waste removal strategies, Gan Cheng et al. [34,35] integrated experimental studies with molecular dynamics simulations to gain deeper insights into microscopic interactions within the system, presenting a novel approach for the efficient separation of nitric acid and acetic acid from waste liquids.

The main objective of this study is to explore the separation performance of the TBP extraction system for mixed solutions of nitric acid and acetic acid under varying operating conditions. By analyzing the impact of factors such as initial acid concentrations, phase ratio, TBP concentration, and the number of extraction stages on the distribution ratios of nitric acid and acetic acid, this study aims to optimize the TBP extraction process and provide theoretical and technical support for industrial-scale acidic wastewater treatment.

The focus of this research is simulating the concentration levels of nitric acid and acetic acid typically found in the Purex process to investigate the selective distribution behavior of TBP for these acids. Furthermore, this study holds significant implications for environmental protection and resource recovery. The discharge of acid-containing wastewater poses environmental hazards and results in the loss of valuable chemical resources [36]. Efficient separation and recovery of nitric acid and acetic acid from mixed solutions can help reduce pollution and generate economic benefits for industries.

## 2. Results and Discussion

### 2.1. Effect of Initial Nitric Acid Concentration

**Figure 1:**
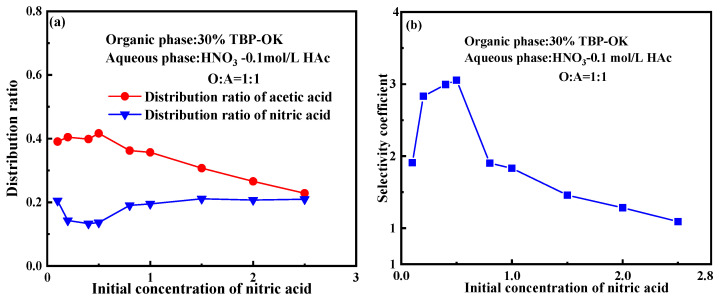
Effect of initial nitric acid concentration on the extraction behavior of nitric acid and acetic acid. (**a**) Influence of nitric acid concentration on the distribution ratio; (**b**) influence of nitric acid concentration on the selectivity coefficient. (Experimental conditions: organic phase: 30% TBP-OK; aqueous phase: 0.1 mol/L HAc-HNO_3_; O/A ratio = 1:1; contact time: 10 min).

The extraction behavior of nitric acid and acetic acid was investigated in a 30% TBP/OK system under varying concentrations of nitric acid in the aqueous phase (0.1–2.5 M). Figure 1 shows the distribution behavior of nitric acid and acetic acid when the concentration of acetic acid in the aqueous phase is held constant at 0.1 M.

As depicted in Figure 1a, the distribution ratio of nitric acid shows noticeable changes at lower concentrations (e.g., from 0.1 M to 0.5 M), with a slight decreasing trend from 0.205 to 0.133. As the concentration of nitric acid increases further, the distribution ratio gradually stabilizes, remaining approximately 0.2 at higher concentrations (e.g., above 1.0 M). This indicates that the distribution behavior of nitric acid reaches equilibrium at higher concentrations, potentially due to the saturation effect of the extractant or competitive interactions among molecules. As the concentration of nitric acid increases, its concentration in the aqueous phase approaches its saturation point, limiting further solubility [37]. When the aqueous phase nears saturation, additional increases in the concentration of nitric acid do not significantly alter its concentration in the aqueous phase, resulting in a stabilization of the distribution ratio.

The distribution ratio of acetic acid gradually decreases as the initial concentration of nitric acid increases, dropping from approximately 0.40 at a low nitric acid concentration to 0.229 at a higher concentration. This trend indicates that nitric acid significantly influences the distribution of acetic acid in the organic phase. The inhibitory effect of nitric acid on the distribution ratio of acetic acid may be attributed to several factors: (1) As the concentration of nitric acid rises, the ionic strength of the aqueous phase increases as well. This can impact the dissociation and coordination behavior of acetic acid, making it more likely to remain in the aqueous phase and thereby reducing its concentration in the organic phase. (2) At a high nitric acid concentration, the increased ionic strength in the aqueous phase can modify the ionization state of acetic acid, further affecting its distribution behavior in the organic phase and leading to a noticeable decrease in its distribution ratio. (3) As the concentration of nitric acid increases, competition for TBP binding sites intensifies. Nitric acid molecules preferentially form complexes with TBP, reducing the chances for TBP to interact with acetic acid. (4) At a high nitric acid concentration, interactions between acetic acid and nitric acid in the aqueous phase may also impact the extraction behavior of acetic acid. Nitric acid, as a strong acid, may form hydrogen bonds or other weak interactions with acetic acid, altering its form in the aqueous phase. These molecular interactions can influence the distribution behavior of acetic acid.

By comparing the distribution ratio trends of acetic acid and nitric acid, it is evident that their distribution behavior in the TBP extraction system differs significantly. While the distribution ratio of nitric acid increases with its concentration, that of acetic acid declines, suggesting a competitive interaction for TBP extraction sites.

As shown in Figure 1b, the selectivity coefficient initially increases and then decreases as the initial concentration of nitric acid rises. This indicates that at a lower acidity level, the selectivity of TBP for acetic acid increases with a higher concentration of nitric acid. However, as the concentration of nitric acid continues to increase, the selectivity of TBP for acetic acid gradually weakens. The decline in the selectivity coefficient can be attributed to the competition for extraction sites and the complex molecular interactions that may arise at a higher nitric acid concentration, such as synergistic effects among multiple ions or the formation of complexes.

**Figure 2:**
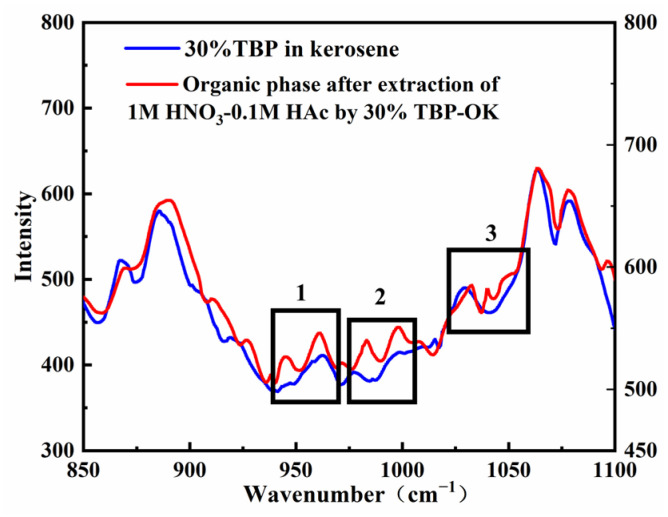
Raman spectra of the organic phase before and after extraction. The boxed regions (1, 2, and 3) highlight notable spectral changes after extraction.

Figure 2 compares the Raman spectra of the organic phase (30% TBP-OK) before and after the extraction of a 1 M HNO_3_-0.1 M HAc solution at a 1:1 phase ratio. The spectral region corresponding to the P=O double bond vibration shows an increase in intensity in the red curve compared to the blue curve, indicating a change in the chemical environment of the P=O bond after extraction. This change is likely due to the formation of complexes between TBP and nitric acid or acetic acid, which alters the vibration mode of the P=O bond.

The characteristic Raman peak for acetic acid appears at approximately 930 cm^−1^ [38], corresponding to C=O stretching vibration, while the characteristic peak for nitric acid typically appears near 1045 cm^−1^ [39], associated with N=O bond vibration. After extraction, small peaks are observed around 983 cm^−1^ and 1040 cm^−1^ in the organic phase, suggesting interactions between TBP molecules and nitric or acetic acid that affect the molecular vibration mode of TBP. However, due to signal overlap in this region and the low concentration of acids extracted into the organic phase, definitively attributing these peaks to specific contributions from nitric acid or acetic acid is challenging.

### 2.2. Effect of Initial Acetic Acid Concentration

**Figure 3:**
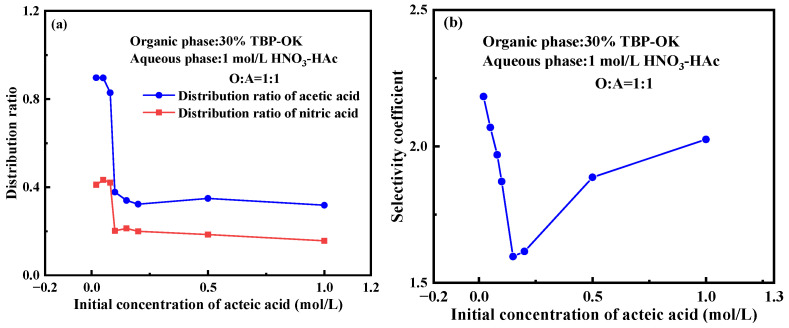
Impact of initial acetic acid concentration on the extraction of nitric acid and acetic acid. (**a**) Effect of acetic acid concentration on the distribution ratio; (**b**) effect of acetic acid concentration on the selectivity coefficient. (Experimental conditions: organic phase: 30% TBP-OK; aqueous phase: 1 mol/L HNO_3_-HAc; O/A ratio = 1:1; contact time: 10 min).

The extraction behavior was examined in a 30% TBP-OK with varying concentrations of acetic acid (0–1.0 M) in the aqueous phase. As depicted in Figure 3a, at low initial concentrations of acetic acid (0.00 M to 0.05 M), the distribution ratio of acetic acid is relatively high, peaking at 0.897 when the initial concentration is 0.020 M. This indicates that acetic acid tends to partition into the organic phase. In the moderate concentration range (0.05–0.20 M), the distribution ratio of acetic acid gradually decreases, reaching 0.323 at an initial concentration of 0.200 M. This suggests that acetic acid begins to favor the aqueous phase, with its solubility in the organic phase diminishing. This shift could be due to an increase in hydrogen bonding within the aqueous phase at these concentrations, causing more acetic acid to remain in the aqueous phase. As the acetic acid concentration continues to increase, the distribution ratio remains relatively low. This indicates that although the organic phase can still absorb a certain amount of acetic acid, the overall extraction efficiency remains low. This behavior might be related to the saturation of TBP extraction capacity and the increasing affinity of acetic acid for the aqueous phase.

At low initial concentrations of acetic acid (<0.05 M), the distribution ratio of nitric acid remains relatively high, ranging from 0.4 to 0.5. This indicates that at low acetic acid concentrations, nitric acid can be distributed relatively evenly between the organic and aqueous phases, with the organic phase showing a strong capacity for absorbing nitric acid. As the concentration of acetic acid increases, the distribution ratio of nitric acid gradually decreases, reaching a minimum of 0.200. This behavior may be attributed to competitive interactions of acetic acid in the organic phase: as its concentration rises, acetic acid molecules occupy more space in the organic phase, inhibiting the migration of nitric acid. When the acetic acid concentration reaches 0.500 M and 1.000 M, the distribution ratio of nitric acid stabilizes at a relatively low level. The decreasing distribution ratio of nitric acid as acetic acid concentration increases suggests that the presence of acetic acid in the organic phase suppresses the distribution of nitric acid. This may be related to the solute repulsion effect or coordination interactions within the organic phase.

The selectivity coefficient reflects the competitive extraction dynamics of acetic acid and nitric acid in the organic phase. As shown in Figure 3b, at a low acetic acid concentration, the selectivity coefficient is high (around 2.1). As the concentration of acetic acid increases, the selectivity coefficient generally declines, reaching 1.871 at 0.100 M, indicating that the selectivity for acetic acid weakens within this concentration range. This may be due to the limited solubility of the organic phase, which cannot accommodate more acetic acid, while also impacting the distribution of nitric acid. At higher concentrations of acetic acid (0.50–1.00 M), the selectivity coefficient exhibits a slight increase, reaching 2.025 at 1.000 M. This could be attributed to enhanced molecular association among acetic acid molecules in the organic phase, which promotes their extraction.

### 2.3. Effect of Phase Ratio

**Figure 4:**
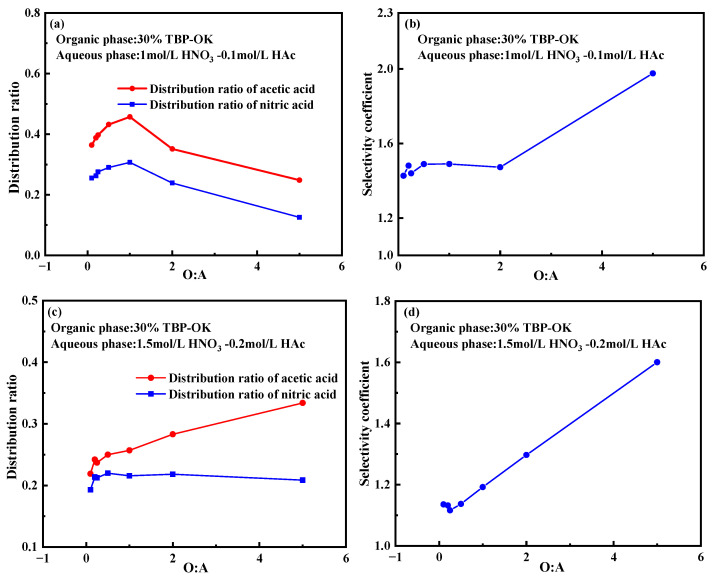
Impact of phase ratio on the extraction of nitric acid and acetic acid. (**a**) Effect of phase ratio on the distribution ratio. (**b**) Effect of phase ratio on the selectivity coefficient. (**c**) Effect of phase ratio on the distribution ratio. (**d**) Effect of phase ratio on the selectivity coefficient. Experimental conditions: (**a**,**b**) (organic phase: 30% TBP-OK; aqueous phase: 1 mol/L HNO_3_-0.1 mol/L Hac; contact time: 10 min); (**c**,**d**) (organic phase: 30% TBP-OK; aqueous phase: 1.5 mol/L HNO_3_-0.2 mol/L HAc; contact time: 10 min).

The extraction behavior in 30% TBP-OK was studied under varying organic-to-aqueous phase ratios (O:A). As shown in Figure 4a, the distribution ratio of nitric acid is highest with a value of 0.307 at an O:A ratio of 1:1. When the phase ratio shifts to 1:2, 1:4, 1:5, and 1:10, the distribution ratio of nitric acid gradually decreases, reaching a minimum of 0.255. Conversely, as the proportion of the organic phase increases (e.g., O:A = 2:1 and O:A = 5:1), the distribution ratio of nitric acid continues to decline. This suggests that higher organic phase proportions dilute the extraction capacity of the solvent, resulting in a lower concentration of nitric acid in the organic phase. Thus, increasing the proportion of the organic phase does not favor the distribution of nitric acid.

A similar trend is observed for the distribution ratio of acetic acid. At an O:A ratio of 1:1, the distribution ratio of acetic acid is 0.457, the highest value recorded. As the proportion of the aqueous phase increases (e.g., O:A = 1:2, 1:4, 1:5, and 1:10), the distribution ratio of acetic acid gradually decreases, reaching a minimum of 0.364. When the proportion of the organic phase further increases (e.g., at ratios of 2:1 and 5:1), the distribution ratio of acetic acid drops significantly.

At O:A = 1:1, the selectivity coefficient is relatively low, as both acids exhibit their highest distribution ratios, indicating that the system shows similar affinity for both acids. As the proportion of the organic phase increases, the selectivity coefficient rises, indicating that TBP demonstrates stronger selectivity for acetic acid under higher organic phase conditions.

The extraction behavior of 1.5 mol/L HNO_3_ and 0.2 mol/L HA_C_ in a 30% TBP-OK system was also investigated under different phase ratios (O:A). As shown in Figure 4c, the distribution ratio of nitric acid remains relatively stable across the range tested, maintaining values between 0.1 and 0.15. Even with an increased phase ratio, the distribution ratio of nitric acid does not significantly change, indicating low extraction efficiency in the TBP system. This may be due to its strong polarity and its high solubility in the aqueous phase [40]. In contrast, the distribution ratio of acetic acid increases as the phase ratio rises, suggesting that acetic acid has a higher distribution ratio in the TBP-OK system and is more affected by the phase ratio. This increase may be attributed to the molecular structure of acetic acid and its better solubility in the organic phase [41].

Figure 4d indicates that as the O:A increases, the selectivity coefficient rises in an almost linear manner. At an O:A ratio of 4–5, the selectivity coefficient approaches 1.5, indicating a significantly higher selectivity for acetic acid over nitric acid. This trend demonstrates that at higher phase ratios, the TBP-OK shows improved extraction capability for acetic acid, while the impact on nitric acid remains limited. This could be due to the lower polarity of acetic acid, which allows it to be more readily extracted into the hydrophobic organic phase. At higher phase ratios, the organic phase provides greater capacity to accommodate acetic acid, thus enhancing its extraction.

Comparing the trends in parameter changes between conditions of 1.5 M HNO_3_-0.2 M HAc and those of 1 M HNO_3_-0.1 M HAc reveals that at a higher initial nitric acid concentration, the distribution ratio remains stable and is less influenced by changes in phase ratio. This suggests that higher initial concentrations of nitric acid contribute to a more stable distribution behavior within the system.

### 2.4. Effect of TBP Concentration

**Figure 5:**
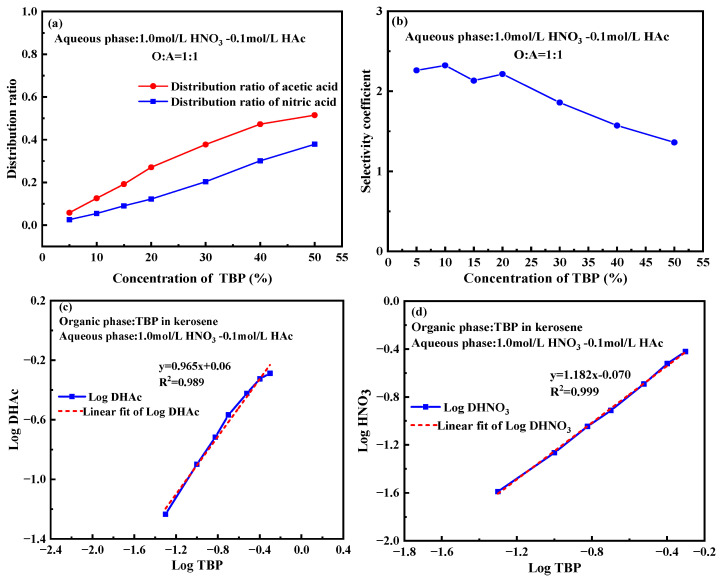
Impact of TBP content on the extraction of nitric acid and acetic acid. (**a**) Effect of TBP content on the distribution ratio. (**b**) Effect of TBP content on the selectivity coefficient. (**c**) Log plot of TBP content vs. distribution ratio for acetic acid. (**d**) Log plot of TBP content vs. distribution ratio for nitric acid. (Experimental conditions: organic phase with varying TBP content in kerosene; aqueous phase: 1 mol/L HNO_3_-0.1 mol/L HAc; O:A = 1:1; contact time: 10 min).

The extraction behavior of nitric acid and acetic acid was investigated using different TBP concentrations in kerosene, with the aqueous phase containing 1 mol/L HNO_3_ and 0.1 mol/L HAc. As illustrated in Figure 5a, the equilibrium concentrations of nitric acid and acetic acid in the organic phase show a clear dependence on TBP concentration. As the TBP concentration in the organic phase increased from 5% to 50%, the distribution ratio of nitric acid gradually rose from 0.02 to nearly 0.4, while that of acetic acid increased from approximately 0.1 to nearly 0.6. This indicates that the higher number of polar groups provided by TBP enhances its ability to extract acidic substances, likely due to stronger intermolecular interactions and greater extractant capacity. A higher TBP concentration means more available extraction sites in the organic phase, which improves its capacity to hold acids. This property is particularly useful in industrial applications, such as in the nuclear industry, where selective separation of specific acids from mixed acid solutions is essential. Increasing TBP concentration can significantly enhance the extraction efficiency of acetic acid while having a relatively smaller effect on nitric acid, making the TBP-OK system well suited for selectively extracting acetic acid from mixed solutions.

Figure 5b shows that the selectivity coefficient fluctuates initially but exhibits an overall decreasing trend as TBP concentration increases. As TBP concentration rises from 0% to 50%, the selectivity coefficient drops from nearly 3 to approximately 1.5. This trend indicates that higher TBP content weakens the selectivity for acetic acid while enhancing its relative selectivity for nitric acid. This occurs because, at higher extractant concentrations, the capacity of organic phase for acid molecules approaches saturation. For the less polar acetic acid, additional TBP molecules contribute less selectively as TBP concentration increases, reducing the extraction advantage for acetic acid [40]. At lower TBP concentrations, acetic acid dissolves more easily in the highly hydrophobic organic phase. However, as TBP concentration increases, the organic phase becomes more polar, favoring the extraction of more polar nitric acid and reducing the selectivity for acetic acid [42].

To determine the extraction stoichiometry of TBP, Figure 5c,d presents the logarithmic plot of the distribution coefficients of acetic acid and nitric acid as a function of TBP content in a 1 mol/L HNO_3_-0.1 mol/L HAc system. Linear fitting of the data produced slope values of 0.965 and 1.182, indicating that 1 mole of TBP extracts nearly an equivalent mole of acetic acid and nitric acid. This suggests a strong correlation between TBP concentration and its extraction capacity for both acids.

**Figure 6:**
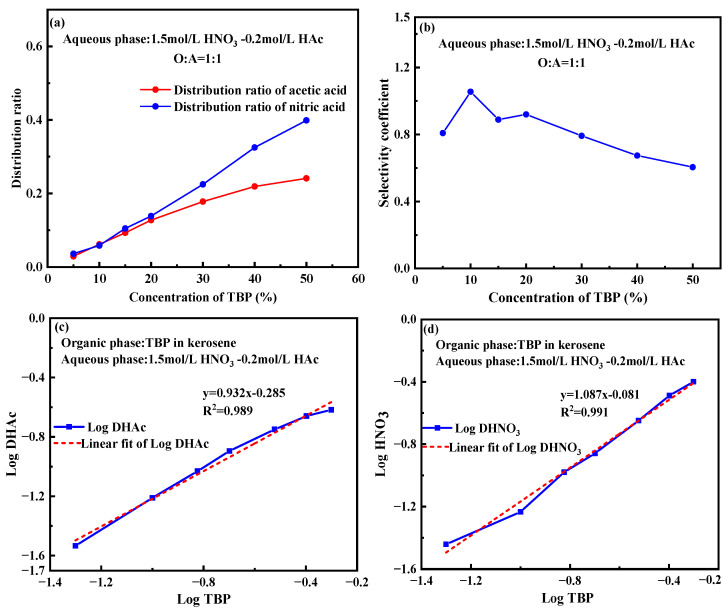
Impact of TBP content on the extraction of nitric acid and acetic acid. (**a**) Effect of TBP content on the distribution ratio. (**b**) Effect of TBP content on the selectivity coefficient. (**c**) Log plot of TBP content vs. distribution ratio for acetic acid. (**d**) Log plot of TBP content vs. distribution ratio for nitric acid. (Experimental conditions: organic phase with varying TBP content in kerosene; aqueous phase: 1.5 mol/L HNO_3_-0.2 mol/L HAc; O:A = 1:1; contact time: 10 min).

To further explore the impact of TBP content on the extraction behavior of nitric acid and acetic acid, the aqueous phase concentration was increased to 1.5 mol/L HNO_3_-0.2 mol/L HAc and brought into contact with TBP-OK containing varying TBP content, followed by oscillatory mixing for extraction. The results shown in Figure 6 indicate that as the TBP concentration increases from 0 to 50%, the distribution ratios of both nitric acid and acetic acid increase linearly, consistent with the trends observed in Figure 5. Notably, TBP concentration has a greater effect on the extraction of nitric acid than on acetic acid, suggesting that as TBP concentration rises, its extraction capacity for nitric acid is enhanced. This is likely due to the increase in available extraction sites, which facilitates the transfer of nitric acid into the organic phase.

In Figure 6b, the selectivity coefficient initially increases at lower TBP concentrations (e.g., 5–10%), reaching a peak of approximately 1.1. However, as the TBP concentration continues to increase, the selectivity coefficient gradually decreases, approaching 0.6 at 50% TBP. This trend indicates that at higher TBP concentrations, the system exhibits greater selectivity for nitric acid extraction.

Compared to the system with 1 mol/L HNO_3_-0.1 mol/L HAc, the distribution ratio of nitric acid consistently increases with rising TBP content in both systems. The higher distribution ratio of nitric acid in the 1.5 mol/L HNO_3_ system indicates that an increase in nitric acid concentration can enhance the extraction performance of TBP.

To further confirm the extraction stoichiometry of TBP, Figure 6c,d present the logarithmic plots of the distribution coefficients of acetic acid and nitric acid as a function of TBP content in a 1.5 mol/L HNO_3_-0.2 mol/L HAc. Linear regression of the data produced slope values of 0.932 for acetic acid and 1.087 for nitric acid, indicating that 1 mole of TBP extracts nearly an equivalent mole of each acid. This result is consistent with the findings shown in Figure 5, reinforcing the reliability of the experimental data.

Furthermore, as nitric acid is a strong acid that fully ionizes in aqueous solutions, an analysis of the logarithmic plots and Raman spectra suggests that the extraction reaction of nitric acid with TBP can be represented as follows:

H^+^_aq_ + NO_3_^−^_aq_ + TBP_org_ → [TBP·HNO_3_]_org_ (1)

This reaction indicates that TBP forms a complex with nitric acid during the extraction process. The equilibrium constant for the extraction reaction can be defined as follows:

(2) $$K_{HNO_{3}}=\frac{\left[TBP\cdot HNO_{3}\right]org}{{\left[H^{+}\right]}_{aq}\times {\left[N{O_{3}}^{-}\right]}_{aq}\times {\left[TBP\right]}_{org}}$$

where $\left[TBP\cdot HNO_{3}\right]org$ is the concentration of the *TBP-HNO*_3_ complex in the organic phase; ${\left[H^{+}\right]}_{aq}$ is the concentration of hydrogen ions in the aqueous phase; ${\left[N{O_{3}}^{-}\right]}_{aq}$ is the concentration of nitrate ions in the aqueous phase; ${\left[TBP\right]}_{org}$ is the concentration of free TBP in the organic phase.

(3) $$D_{HNO_{3}}=\frac{\left[TBP\cdot HNO_{3}\right]org}{{\left[H^{+}\right]}_{aq}\times {\left[N{O_{3}}^{-}\right]}_{aq}}$$

Substituting $D_{HNO_{3}}$ into the Equation (2) for the equilibrium constant and taking the logarithm yields the following:

(4) $$logD_{HNO_{3}}=logK_{HNO_{3}}+log{\left[TBP\right]}_{org}$$

Therefore, the intercept obtained from the logarithmic plot corresponds to $logK_{HNO_{3}}$. For the system of 1.0 mol/L HNO_3_-0.1 mol/L HAc, $logK_{HNO_{3}}$ = −0.070, and $K_{HNO_{3}}=0.851$. For the system of 1.5 mol/L HNO_3_-0.2 mol/L HAc, $logK_{HNO_{3}}$= −0.081, and $K_{HNO_{3}}=0.830$.

**Figure 7:**
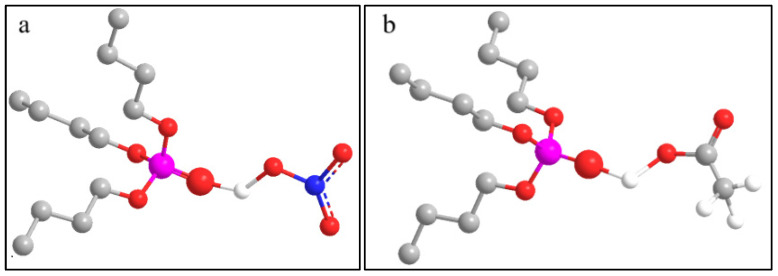
The structure of the complex. (**a**) TBP-HNO_3_; (**b**) TBP-CH_3_COOH. (The colors corresponding to different atoms are as follows: purple: phosphorus, red: oxygen, gray: carbon, blue: nitrogen, white: hydrogen. The dotted lines indicate π bonds. Additionally, for clarity, hydrogen atoms in the TBP structure are omitted).

The phosphate–oxygen double bond (P=O) in the TBP molecule exhibits strong nucleophilic properties, enabling it to form complexes with acids through hydrogen bonding or other types of molecular interactions. The complex structure formed by TBP and HNO_3_/HAc is shown in Figure 7.

### 2.5. Extraction Performance and Mass Transfer in Bench-Scale Extraction

A 16-stage mixer–settler system was employed to enhance interphase mass transfer through multistage mixing. The aqueous phase simulated the composition of nitric acid–acetic acid waste in the Purex process at a concentration of 1 mol/L HNO_3_-0.1 mol/L HAc, while the organic phase consisted of 30% TBP-OK. The operational parameters included a phase ratio of O:A = 1:1, a flow rate of 2.5 mL/min, and a stirring speed of 1200 rpm, using a multistage countercurrent extraction configuration. Each stage in the mixer–settler system was stirred to ensure thorough contact between the organic and aqueous phases, facilitating effective mass transfer.

**Figure 8:**
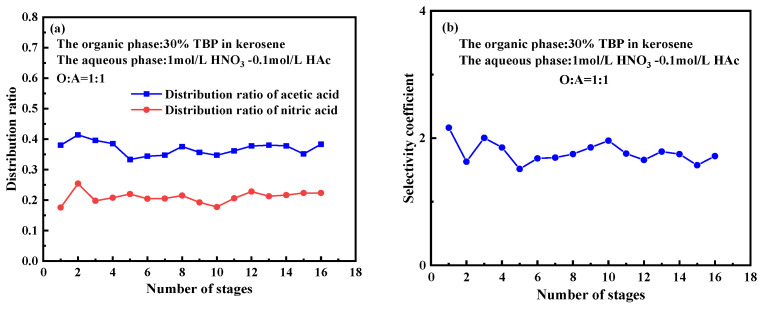
Extraction performance of nitric acid and acetic acid at equilibrium conditions in the bench-scale experiment. (**a**) Distribution ratios at equilibrium conditions for each stage. (**b**) Selectivity coefficients at equilibrium conditions for each stage. (Experimental conditions: organic phase consisting of 30% TBP-OK; aqueous phase: 1.0 mol/L HNO_3_-0.1 mol/L HAc; O:A = 1:1; stirring speed: 1200 rpm; continuous operation time: 5 h).

As shown in Figure 8a, the distribution ratio of acetic acid and nitric acid followed a clear pattern. The distribution ratio of acetic acid ranged from 0.33 to 0.414, indicating a relatively high and stable extraction efficiency across different concentrations of the organic and aqueous phases. The distribution ratio of nitric acid varied between 0.17 and 0.25, which was slightly lower, suggesting that TBP has a comparatively weaker extraction capability for nitric acid.

Throughout the 16 stages, the equilibrium concentrations of acetic acid and nitric acid in both phases exhibited minor fluctuations but remained generally stable, indicating that the system had reached an approximate steady state. These fluctuations suggest that, while each stage tends to reach equilibrium, variations in molecular diffusion rates and limited reaction time can cause concentration changes in certain stages, potentially due to interstage mass transfer resistance.

The equilibrium at higher stages showed that the distribution balance gradually stabilized, indicating the establishment of a dynamic equilibrium between solute transfer and distribution, which is essential for continuous extraction processes. As the number of stages increased, the equilibrium concentration of acetic acid in the organic phase showed a slight increase, likely due to the gradual accumulation of acetic acid in the organic phase as the stages progressed.

The values and trends of the purification coefficient can be used to optimize the concentration of TBP, the organic phase flow and the operating temperature in the countercurrent extraction experiment, so as to achieve efficient separation of the target acid. The purification coefficient is defined as follows:

(5) $$K_{P}=\frac{C_{HAc,initial}/C_{HNO_{3},initial}}{C_{HAc,inst}/C_{HNO_{3},inst}}$$

where $K_{P\;}$ represents the purification coefficient. $C_{HAc,initial}$ is the initial concentration of HAc in the aqueous phase, and $C_{HNO_{3},initial}$ is the initial concentration of HNO_3_ in the aqueous phase. $C_{HAc,inst}$ represents the instantaneous concentration of HAc in the aqueous phase, and $C_{HNO_{3},inst}$ represents the instantaneous concentration of HNO_3_ in the aqueous phase.

**Figure 9:**
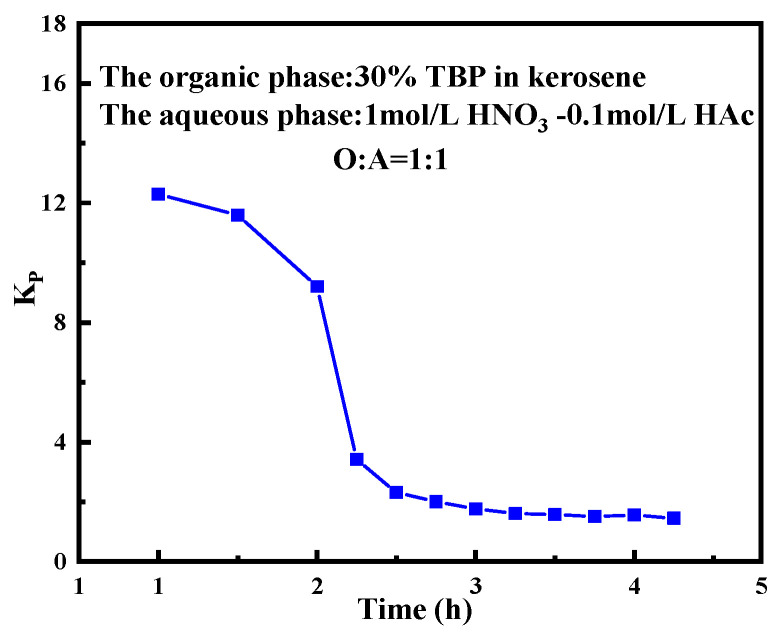
Purification coefficient of instantaneous samples after 16-stage extraction in the bench-scale experiment. Experimental conditions: organic phase composed of 30% TBP-OK; aqueous phase: 1.0 mol/L HNO_3_-0.1 mol/L HAc; O:A = 1:1; stirring speed: 1200 rpm; continuous operation time: 4.25 h.

Figure 9 depicts the variation in the purification coefficient of acetic acid over time, based on the samples collected at different intervals. As shown, the $K_{P}$ experiences a sharp decline during the initial stage (0~2 h), dropping from a high initial value of approximately 12 to around 2.3. After this rapid decrease, the decreasing trend of purification coefficient tends to moderate, and the purification efficiency gradually diminishes. By the 3 h mark, the $K_{P}$ stabilizes at roughly 1.5.

This trend indicates that acetic acid undergoes rapid redistribution between the organic and aqueous phases in the early stage, with the process slowing as the system approaches equilibrium. The initial rapid decline can be attributed to the significant concentration gradient and distribution-driving forces present in the system. During the early phase of extraction, the substantial concentration difference (driving force) between the aqueous phase and organic phase facilitates the quick transfer of acetic acid into the organic phase. Over time, the concentration gradient between the phases decreases, reducing the mass transfer, and the system gradually reaches equilibrium. According to the Nernst distribution law [43], when the concentration of acetic acid in the aqueous and organic phases reaches equilibrium, the distribution process is considered complete.

These experimental results provide valuable guidance for the treatment of acidic wastewater in the industry. By fine-tuning the concentration of TBP and adjusting the phase ratio, selective extraction of the target acidic level can be achieved. For instance, in the practical application, employing higher concentration of TBP and increasing the number of multistage countercurrent extraction stages can enhance the distribution ratio of nitric acid while the distribution ratio of acetic acid remains relatively unchanged or decreases. This strategy enables the efficient separation of nitric acid and acetic acid from acidic wastewater, thereby improving the effectiveness of the treatment process.

In the mass transfer kinetic model, the transfer of acetic acid is influenced by factors such as the diffusion coefficient, interfacial contact area, and stirring rate. Given the high solubility of acetic acid in water, the mass transfer from the organic phase to the aqueous phase tends to be rapid. Under these conditions, the mass transfer process can be approximated by the Fick diffusion model [44], where the transfer rate at each stage is primarily driven by the concentration gradient, with interfacial reactions playing a minor role. It is assumed that each stage approaches local equilibrium, with interphase mass transfer occurring between successive stages.

(6) $$N=K\times \left(C_{A,eq}-C_{A,actual}\right)$$

In this model, *N* represents the mass transfer rate (mol/s), which is the amount of solute transferred across the interface per unit time. *K* denotes the overall mass transfer coefficient, $C_{A,eq}$ is the concentration of nitric acid or acetic acid in both phases at equilibrium, and $C_{A,actual}$ is the concentration measured during the experiment.

Using this model, the concentration difference $\Delta C=C_{A,aq}-C_{A,org}$ can be used to approximate the mass transfer rate *N*, assuming that the mass transfer rate between stages reaches a local equilibrium. The relationship can be expressed as follows:

(7) N=Q×ΔC

where Q is the volumetric flow rate. The results, shown in Table 1, indicate that from stages 1 to 15, the mass transfer rate for acetic acid remains within a narrow range of 1.68 × 10^−6^ to 2.93 × 10^−6^ mol/s, suggesting consistent transfer across these stages. This stability implies that the mass transfer rate for acetic acid is relatively uniform throughout the system. At stage 16, the concentration difference for acetic acid becomes negative, leading to a negative mass transfer rate N_HAc_. This suggests that acetic acid is moving back from the organic phase to the aqueous phase, likely due to local concentration fluctuations or the system nearing equilibrium. For nitric acid, the mass transfer rate fluctuates between 3.05 × 10^−5^ and 3.64 × 10^−5^ mol/s, higher than that of acetic acid. This difference may be attributed to the stronger affinity of nitric acid for the aqueous phase, making its mass transfer rate more responsive to concentration differences between the aqueous and organic phases.

## 3. Materials and Methods

### 3.1. Materials

The organic phase was prepared using tributyl phosphate (TBP, 99% purity, Sinopharm Chemical Reagent Co., Ltd., Shanghai, China) as the extractant, with kerosene as the diluent. The aqueous phase consisted of a nitric acid–acetic acid solution, prepared by diluting concentrated nitric acid (65–68% purity, analytical grade, Sinopharm Chemical Reagent Co., Ltd., Shanghai, China) and glacial acetic acid (analytical grade, Sinopharm Chemical Reagent Co., Ltd., Shanghai, China) with deionized water.

For ion chromatographic analysis, sodium hydroxide (Sinopharm Chemical Reagent Co., Ltd., Shanghai, China) was used as the eluent. Calibration curves were established using standard nitric acid (1000 μg/mL) and acetic acid (1000 μg/mL) solutions.

### 3.2. Methods

**Figure 10:**
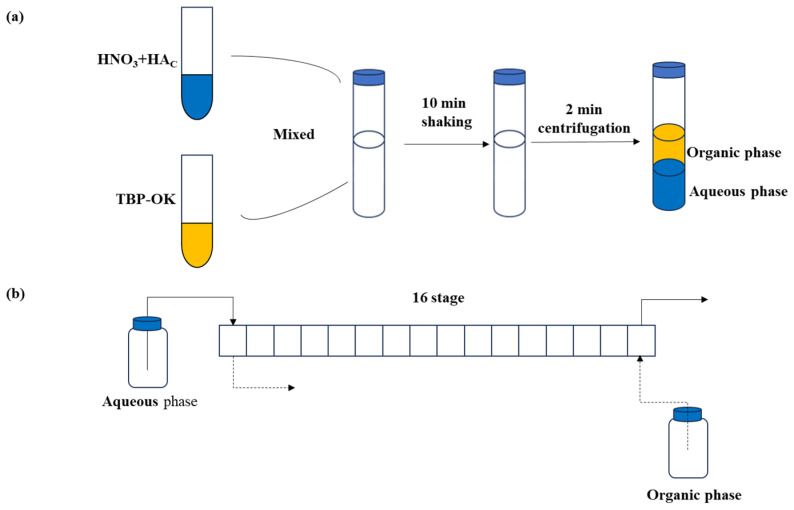
Experimental flow chart. (**a**) Single-stage extraction experiment; (**b**) bench-scale extraction experiment. (The blue color represents the aqueous phase, yellow represents the organic phase, the solid line arrow indicates the flow of the aqueous phase, and the dashed line arrow represents the flow of the organic phase).

This study consists of single-stage experiment and bench-scale extraction. Figure 10 presents a flowchart illustrating the experimental workflow.

#### 3.2.1. Single-Stage Extraction

Single-stage extraction experiments were conducted using the TBP extraction method to investigate the distribution behavior of nitric acid and acetic acid. The experiments were performed at room temperature (25 °C) with an organic-to-aqueous phase ratio (O/A) of 1:1. The organic phase consisted of a 30% TBP-OK solution, while the aqueous phase was a mixture of nitric acid and acetic acid. The two phases were stirred for 10 min, followed by centrifugation for 2 min to facilitate phase separation. The final concentrations of nitric acid and acetic acid in both phases were then analyzed.

#### 3.2.2. Bench-Scale Extraction Experiment

A 16-stage countercurrent extraction experiment was conducted using a mixer system to simulate continuous extraction (Figure S1). Each mixing chamber had a 5 mL volume, with a residence time of 1 min per stage. The aqueous phase consisted of 1 mol/L nitric acid and 0.1 mol/L acetic acid, while the organic phase was 30% TBP-OK. The flow rate was 2.5 mL/min, with an O/A ratio of 1:1, across 16 extraction stages, with stage 0 designated for washing.

At the end of the experiment, instantaneous samples were collected from each outlet for analysis. Additionally, samples were collected at 5 min intervals during the first 30 min of operation, followed by 20 min intervals for both aqueous and organic phases during the remaining operation time.

### 3.3. Analytical Methods

The concentration of acetic acid was determined using ion chromatography. Due to the relatively low distribution ratio of acetic acid, its concentration in the organic phase is low, while it remains higher in the aqueous phase after extraction equilibrium is reached. Therefore, only the concentration of acetic acid in the organic phase was measured, and the concentration in the aqueous phase was calculated using mass balance principles. To measure the concentration of acetic acid in the organic phase, it was back-extracted three times using deionized water, transferred into the aqueous phase, diluted to an appropriate level, and then analyzed by ion chromatography. The ion chromatogram of the acetic acid and nitric acid is presented in Figure S2. Chromatograms for different concentrations of acetic acid were obtained, and a standard curve was established based on the peak area and concentration, as shown in Figure S3. It can be observed that this method provides good linearity for the determination of acetic acid.

The concentration of nitric acid was determined by titration using standard NaOH solution (0.05 M) with phenolphthalein as the indicator. The endpoint was noted when the color changed from colorless to pink, and the readings were recorded to calculate the extraction efficiency. Similarly to acetic acid, the concentration of nitric acid in the organic phase is low, while it is higher in the aqueous phase. Therefore, only the concentration of nitric acid in the organic phase was measured, and the concentration in the aqueous phase was calculated using mass balance.

It should be noted that acetic acid is also present as an acid in the system and can be titrated using NaOH. The total concentration of acetic acid and nitric acid in the organic phase was titrated with phenolphthalein as the indicator [45,46,47], and the concentration of acetic acid (determined by ion chromatography) was subtracted to obtain the concentration of nitric acid.

The distribution ratio of nitric acid ($D_{HNO_{3}}$) is defined as the ratio of the concentration of nitric acid in the organic phase to that in the aqueous phase. It is a critical indicator of the distribution behavior of nitric acid in the water–organic phase system. The formula is as follows [48]:

(8) $$D_{HNO_{3}}=\frac{{\left[HNO_{3}\right]}_{org}}{{\left[HNO_{3}\right]}_{aq}}$$

where ${\left[HNO_{3}\right]}_{org}$ and ${\left[HNO_{3}\right]}_{aq}$ denote the concentrations of nitric acid in the organic phase and aqueous phase, respectively.

The distribution ratio of acetic acid ($D_{CH_{3}COOH}$) is defined as the ratio of the concentration of acetic acid in the organic phase to its concentration in the aqueous phase. The formula is as follows:

(9) $$D_{CH_{3}COOH}=\frac{{\left[CH_{3}COOH\right]}_{org}}{{\left[CH_{3}COOH\right]}_{aq}}$$

where ${\left[CH_{3}COOH\right]}_{org}$ and ${\left[CH_{3}COOH\right]}_{aq}$ denote the concentrations of acetic acid in the organic phase and aqueous phase, respectively.

The selectivity coefficient (*S*) is used to measure the selectivity of the extraction process for a specific component, reflecting the relative selectivity of TBP for acetic acid compared to nitric acid. The formula is as follows:

(10) $$S=\frac{D_{CH_{3}COOH}}{D_{HNO_{3}}}$$

Raman spectroscopy (Raman spectrometer, HORIBA FRANCE SAS, Paris, France) was employed to acquire spectral data of the organic samples before and after extraction.

## 4. Conclusions

This study systematically examines the selective extraction of nitric acid and acetic acid in the Purex process using a TBP-OK system. The key conclusions are as follows:

1. Selective extraction behavior: TBP preferentially extracts acetic acid over nitric acid, especially in the low acid concentration range (HNO₃ < 1 mol/L, HAc < 0.2 mol/L). The selectivity coefficient decreases from 3.05 in a 0.5 mol/L HNO_3_-0.1 mol/L HAc system to 2.18 in a 1 mol/L HNO_3_-0.2 mol/L HAc system.
2. Complex formation and equilibrium: TBP forms stable 1:1 complexes with both nitric acid and acetic acid, with equilibrium constants of approximately 0.85 under typical operating conditions, specifically in a 1.0 mol/L HNO_3_-0.1 mol/L HAc system.
3. Effect of TBP concentration and phase ratio: Increasing TBP concentration enhances nitric acid extraction, while adjusting the phase ratio improves acetic acid separation. Under 1mol/L HNO_3_-0.1mol/L HAc system, a higher phase ratio (5:1) increases the selectivity coefficient to 1.9, while 5% TBP-OK further improves it to 2.27.
4. Process optimization: A 16-stage countercurrent extraction simulation confirms that optimizing TBP concentration and phase ratio effectively enhances the separation of nitric acid and acetic acid, providing a foundation for process improvement in spent fuel reprocessing.

## Figures and Tables

**Table 1:** Mass transfer rate at each stage.

Stages	$\Delta_{HAc}$ (mol/L)	$\Delta_{HNO_{3}}$ (mol/L)	$N_{HAc}$ (mol/s)	$N_{HNO_{3}}$ (mol/s)
1	0.0404	0.731	1.68 × 10^−6^	3.05 × 10^−5^
2	0.0499	0.7449	2.08 × 10^−6^	3.10 × 10^−5^
3	0.055	0.852	2.29 × 10^−6^	3.55 × 10^−5^
4	0.0578	0.8222	2.41 × 10^−6^	3.43 × 10^−5^
5	0.0695	0.8178	2.90 × 10^−6^	3.41 × 10^−5^
6	0.0691	0.8255	2.88 × 10^−6^	3.44 × 10^−5^
7	0.0681	0.8043	2.84 × 10^−6^	3.35 × 10^−5^
8	0.0645	0.812	2.69 × 10^−6^	3.38 × 10^−5^
9	0.0691	0.8286	2.88 × 10^−6^	3.45 × 10^−5^
10	0.0656	0.8742	2.73 × 10^−6^	3.64 × 10^−5^
11	0.0675	0.8183	2.81 × 10^−6^	3.41 × 10^−5^
12	0.0676	0.7754	2.82 × 10^−6^	3.23 × 10^−5^
13	0.0679	0.8198	2.83 × 10^−6^	3.42 × 10^−5^
14	0.0668	0.8092	2.78 × 10^−6^	3.37 × 10^−5^
15	0.0702	0.8135	2.93 × 10^−6^	3.39 × 10^−5^
16	−0.3009	0.8243	−1.25 × 10^−5^	3.43 × 10^−5^

## Data Availability

The original contributions presented in this study are included in the article/Supplementary Materials. Further inquiries can be directed to the corresponding authors.

## Supplementary Materials

The following supporting information can be downloaded at: https://www.mdpi.com/article/10.3390/molecules30081814/s1, Figure S1: Bench scale extraction system installation diagram; Figure S2: Ion chromatogram of nitric acid and acetic acid; Figure S3: Calibration curve for acetic acid ion chromatography.

## Nomenclature

*AHA*	acetohydroxamic acid
${\left[CH_{3}COOH\right]}_{org}$	the concentration of acetic acid in the organic phase
${\left[CH_{3}COOH\right]}_{aq}$	the concentration of acetic acid in the aqueous phase
$C_{A,eq}$	concentration of nitric acid or acetic acid in both phases at equilibrium, mol/L
$C_{A,actual}$	concentration measured during the experiment, mol/L
$C_{A,org}$	concentration of nitric acid or acetic acid in the organic phase, mol/L
$C_{A,aq}$	concentration of nitric acid or acetic acid in the aqueous phase, mol/L
ΔC	the concentration difference between the organic phase and aqueous phase, mol/L
$D_{HNO_{3}}$	the distribution ratio of nitric acid
$D_{CH_{3}COOH}$	the distribution ratio of acetic acid
$HA_{c}$	acetic acid
${\left[HNO_{3}\right]}_{org}$	concentration of nitric acid in the organic phase, mol/L
${\left[HNO_{3}\right]}_{aq}$	concentration of nitric acid in the aqueous phase, mol/L
${\left[H^{+}\right]}_{aq}$	concentration of hydrogen ions in the aqueous phase, mol/L
$K_{P\;}$	the purification coefficient
*K*	overall mass transfer coefficient
${\left[N{O_{3}}^{-}\right]}_{aq}$	concentration of nitrate ions in the aqueous phase
N	the mass transfer rate, mol/s
*Q*	volumetric flow rate, ml/min
S	the selectivity coefficient
TBP	tributyl phosphate
*TBP-OK*	tributyl phosphate in organic kerosene
$\left[TBP\cdot HNO_{3}\right]org$	concentration of the TBP-HNO_3_ complex in the organic phase, mol/L
${\left[TBP\right]}_{org}$	concentration of free TBP in the organic phase

## References

[1] Ekins P. (2004). Step changes for decarbonising the energy system: Research needs for renewables, energy efficiency and nuclear power. Energy Policy.

[2] Francis A.J. (1990). Characteristics of nuclear and fossil energy wastes. Experientia.

[3] Milliken J.A., Joseck F., Wang M., Yuzugullu E. (2007). The advanced energy initiative. J. Power Sources.

[4] Buchao X.U., Yanfei Z., Ming H. (2010). Analysis on development modes and routes of nuclear energy in the context of low carbon economy. Resour. Sci..

[5] Kurniawan T.A., Othman M.H.D., Singh D., Avtar R., Hwang G.H., Setiadi T., Lo W.H. (2022). Technological solutions for long-term storage of partially used nuclear waste: A critical review. Ann. Nucl. Energy.

[6] Salvatores M., Palmiotti G. (2011). Radioactive waste partitioning and transmutation within advanced fuel cycles: Achievements and challenges. Prog. Part. Nucl. Phys..

[7] Kuzmin V.I., Kuzmin D.V., Gudkova N.V., Kalyakin S.N., Mulagaleeva M.A., Alekseenko V.N., Aksyutin P.V., Bartseva Y.V., Ivanov A.V., Kryuchek N.M. (2022). Autocatalytic decomposition of tributyl phosphate in the spent extractant of the PUREX process for safe disposal of radioactive impurities. Hydrometallurgy.

[8] Saab M., Réal F., Šulka M., Cantrel L., Virot F., Vallet V. (2017). Facing the challenge of predicting the standard formation enthalpies of n-butyl-phosphate species with ab initio methods. J. Chem. Phys..

[9] Chen H., Taylor R., Woodhead D., Sarsfield M., Whittaker D., Carrott M., Keywood B., Taylor K., Jobson M., Masters A. (2024). Experimental testing and process simulation of flowsheets for the co-separation of uranium and plutonium using acetohydroxamic acid as a complexing agent. Prog. Nucl. Energy.

[10] Herbst R., Baron P., Nilsson M. (2011). Standard and advanced separation: PUREX processes for nuclear fuel reprocessing. Reviews in Advanced Separation Techniques for Nuclear Fuel Reprocessing and Radioactive Waste Treatment.

[11] Shuangchen M., Jin C., Gongda C., Weijing Y., Sijie Z. (2016). Research on desulfurization wastewater evaporation: Present and future perspectives. Renew. Sustain. Energy Rev..

[12] Digiano F.A., Scaramelli A.B. (1974). Wastewater treatment: Physical and chemical methods. J. Water Pollut. Control Fed..

[13] Agarwal C., Pandey A.K. (2023). Remediation and recycling of inorganic acids and their green alternatives for sustainable industrial chemical processes. Environ. Sci. Adv..

[14] Zhao X., Liu Z., Zhao J., Kang T., Yan C., Ju C., Ma L., Zhang X., Wang Y., Wu Y. (2024). Highly efficient molecular film for inhibiting volatilization of hazardous nitric acid. Environ. Res..

[15] Cantwell F.F., Losier M. (2002). Chapter 11 Liquid-liquid extraction. Comprehensive Analytical Chemistry.

[16] Mazzola P.G., Lopes A.M., Hasmann F.A., Jozala A.F., Penna T.C., Magalhaes P.O., Pessoa A. (2008). Liquid-liquid extraction of biomolecules: An overview and update of the main techniques. J. Chem. Technol. Biotechnol..

[17] Rodrigues G.D., da Silva M.D.C.H., da Silva L.H.M. (2008). Liquid-liquid extraction of metal ions without use of organic solvent. Sep. Purif. Technol..

[18] Moreira V.R., Lebron Y.A.R., Gontijo D., Amaral M.C.S. (2022). One-step recycling of mineral acid from concentrated gold mining wastewater by high-temperature liquid-liquid extraction. Sep. Purif. Technol..

[19] Chadni M., Moussa M., Athès V., Allais F., Ioannou I. (2023). Membrane contactors-assisted liquid-liquid extraction of biomolecules from biorefinery liquid streams: A case study on organic acids. Sep. Purif. Technol..

[20] Ehsan R., Thomas B., Kersten S.R.A., Van H.A.G.J., Boelo S. (2018). Liquid-liquid extraction-based process concepts for recovery of carboxylic acids from aqueous streams evaluated for dilute streams. Chem. Eng. Res. Des..

[21] Shin C.-H., Kim J.-Y., Kim J.-Y., Kim H.-S., Lee H.-S., Mohapatra D., Ahn J.-W., Ahn J.-G., Bae W. (2009). Recovery of nitric acid from waste etching solution using solvent extraction. J. Hazard. Mater..

[22] Karunanithi S., Kapoor A., Kumar P.S., Balasubramanian S., Rangasamy G. (2023). Solvent extraction of acetic acid from aqueous solutions: A review. Sep. Sci. Technol..

[23] Chen F., Wang X., Liu W., Liang B., Yue H., Li C. (2016). Selective extraction of nitric and acetic acids from etching waste acid using N235 and MIBK mixtures. Sep. Purif. Technol..

[24] Shin C.-H., Kim J.-Y., Kim J.-Y., Kim H.-S., Lee H.-S., Mohapatra D., Ahn J.-W., Ahn J.-G., Bae W. (2009). A solvent extraction approach to recover acetic acid from mixed waste acids produced during semiconductor wafer process. J. Hazard. Mater..

[25] Wongsawa T., Koonsang T., Kunthakudee N., Prapasawat T., Maneeintr K., Pancharoen U. (2018). The experimental investigations on viscosity, surface tension, interfacial tension and solubility of the binary and ternary systems for tributyl phosphate (TBP) extractant in various organic solvents with water: Thermodynamic NRTL model and molecular interaction approach. J. Mol. Liq..

[26] Zhou Z., Qin W., Fei W. (2011). Extraction equilibria of lithium with tributyl phosphate in three diluents. J. Chem. Eng. Data.

[27] Shi C., Duan D., Jia Y., Jing Y. (2014). A highly efficient solvent system containing ionic liquid in tributyl phosphate for lithium ion extraction. J. Mol. Liq..

[28] Phillips W.D., Milne N. (2017). Ashless phosphorus–containing lubricating oil additives. Reviews in Lubricant Additives.

[29] Yang X., Yu G., Xu L., Wang J. (2022). Degradation of the mixed organic solvents of tributyl phosphate and n-dodecane by heterogeneous Fenton-like oxidation using nanoscale zero-valent iron as the catalyst. Chemosphere.

[30] Ma J., Wang K., Li M., Liu T., Yang C. (2024). Technological process for treating radioactive TBP/OK spent solvents. J. Phys. Conf. Ser..

[31] Carrot M., Gregson C., Taylor R. (2013). Neptunium extraction and stability in the GANEX solvent: 0.2 M TODGA/0.5 M DMDOHEMA/kerosene. Solvent Extr. Ion. Exch..

[32] Pathak P., Kumari N., Prabhu D., Manchanda V. (2012). Redox behavior of neptunium (V) in tributyl phosphate and N, N-dihexyl octanamide extractants dissolved in n-dodecane. J. Solut. Chem..

[33] Shakya A.K., Jaiswal Y., Pal S.L., Srivastava S. (2022). Reactive extraction of acetic acid by using tri-butyl-phosphate with different diluents. Chem. Data Collect..

[34] Cheng G., Li Y., Cao Y., Zhang Z. (2023). A novel method for the desulfurization of medium–high sulfur coking coal. Fuel.

[35] Cheng G., Li Y., Cao Y., Wang X., Li E., Guo Y., Lau E.V. (2024). New insights on the understanding of sulfur-containing coal flotation desulfurization. Minerals.

[36] Pratap B., Kumar S., Nand S., Azad I., Bharagava R.N., Ferreira L.F.R., Dutta V. (2023). Wastewater generation and treatment by various eco-friendly technologies: Possible health hazards and further reuse for environmental safety. Chemosphere.

[37] Wright A., Paviet-Hartmann P. (2010). Review of physical and chemical properties of tributyl phosphate/diluent/nitric acid systems. Sep. Sci. Technol..

[38] Shen X., Han K., Ma L., Gao M., Xu X., Luo J. (2018). Nano-Ag-forest based surface enhanced Raman spectroscopy (SERS) of confined acetic acid. Colloids Surf. A.

[39] Lucas H., Petitet J.-P. (1999). High pressure Raman spectroscopy of nitric acid. J. Phys. Chem. A.

[40] Kertes A., King C.J. (1986). Extraction chemistry of fermentation product carboxylic acids. Biotechnol. Bioeng..

[41] Marti M.E. (2016). Solvent modification effect on the physical and chemical extraction of acetic acid. Sep. Sci. Technol..

[42] Tamada J.A., Kertes A.S., King C.J. (1990). Extraction of carboxylic acids with amine extractants. 1. Equilibria and law of mass action modeling. Ind. Eng. Chem. Res..

[43] Rydberg J., Choppin G.R., Musikas C., Sekine T. (2004). Solvent extraction equilibria. Reviews in Solvent Extraction Principles and Practice, Revised and Expanded.

[44] Wijmans J.G., Baker R.W. (1995). The solution-diffusion model: A review. J. Membr. Sci..

[45] Karita S., Kaneta T. (2014). Acid–base titrations using microfluidic paper-based analytical devices. Anal. Chem..

[46] Patil A.R., Shastri L.A., Tilakraj T., Inamdar S.R., Shastri S.L., Hebbar N.U., Pawar V., Sunagar V.A. (2022). Synthesis and characterization of acid-base indicator: Exploring pH sensor, photophysical, thermal applications and theoretical study. J. Mol. Struct..

[47] Sadler G.D., Murphy P.A. (2010). pH and titratable acidity. Food Anal..

[48] Bell K., Geist A., McLachlan F., Modolo G., Taylor R., Wilden A. (2012). Nitric acid extraction into TODGA. Procedia Chem..

