# Supplementary material for: A Study on the Separation of Nitric Acid and Acetic Acid from Simulated Reprocessing Waste by TBP Extraction
Source: Molecules. 2025 Apr 17;30(8):1814. doi: 10.3390/molecules30081814 (PMC12029649; doi:10.3390/molecules30081814)
Supplement: Supplementary file 1 [file molecules-30-01814-s001.zip › molecules-3511564-supplementary.pdf]

Supplementary Materials

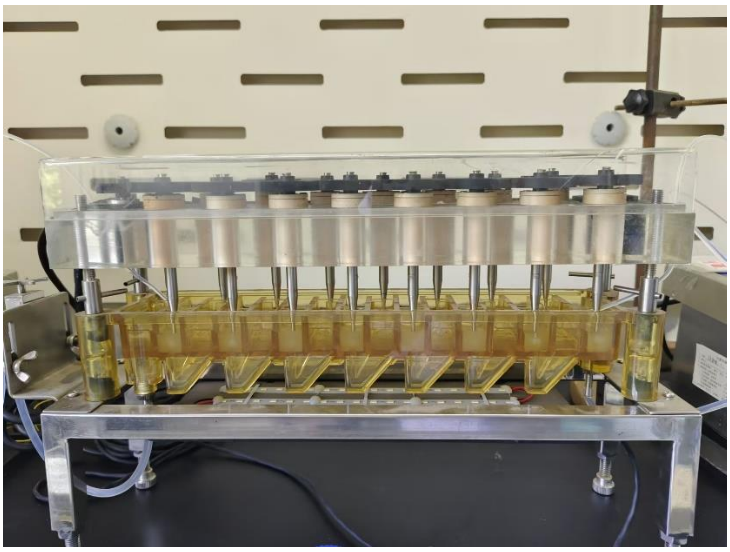

Figure S1. Bench scale extraction system installation diagram

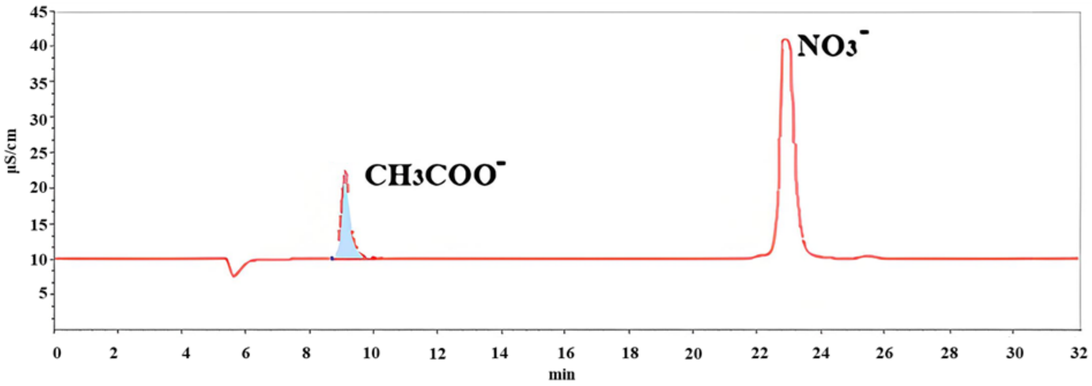

Figure S2. Ion chromatogram of nitric acid and acetic acid.

The ion chromatogram of the acetic acid and nitric acid is presented in Figure S2. As shown, the retention time for acetic acid is 9.1 min, while that for nitric acid is 23 min. The substantial difference in their retention times allows for the accurate determination of acetic acid concentration using ion chromatography.

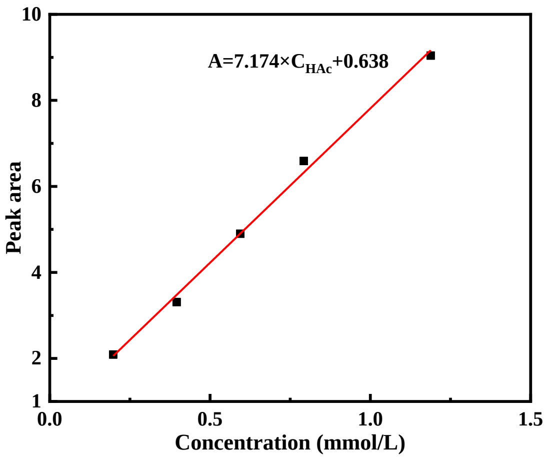

Figure S3. Calibration curve for acetic acid ion chromatography.

Chromatograms for different concentrations of acetic acid were obtained, and a standard curve was established based on the peak area and concentration, as shown in Figure S3. It can be observed that this method provides good linearity for the determination of acetic acid.

Hydrolytic Decomposition of AHA

1. Under acidic conditions, AHA undergoes hydrolysis, forming acetic acid and hydroxylamine (NH_2OH):

$$CH_3CONHOH + H_2O \rightarrow CH_3COOH + NH_2OH$$

2. Further Decomposition of Hydroxylamine (NH_2OH)

Hydroxylamine is unstable in acidic environments and may decompose into nitrous oxide (N_2O) and water:

$$2NH_2OH \rightarrow N_2O + 3H_2O$$

Alternatively, under certain conditions, it can generate ammonia:

$$NH_2OH + H^+ \rightarrow NH_3 + H_2O$$

3. Oxidative Decomposition of AHA

In the presence of strong acids (such as concentrated nitric acid), AHA can undergo oxidative decomposition, producing acetic acid, ammonia, and carbon dioxide:

$$CH_3CONHOH + H_2O \rightarrow CH_3COOH + NH_3 + CO_2$$
